# Professional Advertising

**Published:** 1896-04-18

**Authors:** 


### The Hospital

An Institutional Journal of

The Medical Sciences and Hospital Administration, with

AN EXTRA NURSING SUPPLEMENT.

Vol. XX.—No. 499.] [April 18, 1896.

### Professional Advertising.

"F.R.C.P. and a Young Author" addresses certain queries to the medical profession through the *British Medical Journal*, which are of urgent practical importance to him and to other clever and ambitious young doctors, and which demand the justest and most rational consideration of which the profession, as a whole, is capable. The following are his difficulties: He is publishing a book of a purely medical character, but a book which, in certain parts of it, is of great interest to the general reader. May he, or may he not, under the circumstances, send the book for review to the lay press, and advertise it for sale in the non-professional newspapers? The question is one of great importance from a business point of view, not to "F.R.C.P." only, but also to his publisher, who is bringing out the book under certain defined conditions as to profits, and who will suffer, or be benefited, according as the book is or is not well advertised and extensively sold.

A certain portion of the profession, it seems, have a cut-and-dried answer ready, and that portion numbers in its ranks a good many "Fellows" of "F.R.C.P.'s" college, which, we assume, is the Royal College of Physicians of London. This portion of the profession puts its foot firmly down at once, and plainly says, "No; it is not according to professional etiquette that a medical book should either be sent to the lay press for review or advertised for sale in non-professional newspapers." To this dictum, in turn, "F.R.C.P." has his rejoinder. "If that be so," he says in effect, "how is it that certain seniors of the profession and of the College of Physicians have, as a matter of fact, had their books extensively, not only reviewed in the lay press, but advertised for sale?" And he mentions, among others, the late Sir William Jenner, President of the Royal College of Physicians; Sir Richard Quain, President of the General Medical Council; and Sir George Johnson, F.R.C.P.

Now, "F.R.C.P. and a Young Author" is evidently a perfectly frank and fair-minded person. He sees that there may be grounds, solid and adequate, for restricting the notice and sale of medical books to medical newspapers and medical persons. But he sees also that if such a wall as this is to be built round the profession it cannot be right for senior and eminent physicians to boldly jump over the wall themselves and then to ask their colleges and newspapers to keep the younger and less known doctors strictly and religiously inside it. This we all see, as well as a "Young Author." If it be a professional misdemeanour of a grave kind for a young "F.R.C.P." to send his book for review to the lay press, how can it be anything but a professional misdemeanour of a still graver kind for "F.R.C.P.s" who have grown grey in the recognition of their college to do the same thing?

Here we are clearly at a deadlock. But what seems most strange to many medical men is that not a few members of our profession appear to be quite willing for things to remain at a deadlock. That, however, cannot be. Our profession is great, and it is also learned. We owe it to ourselves, therefore, to get rid of the discredit of such a foolish and injurious condition of things. But, besides, there is a business aspect of the case. A great many young men are coming into the profession who have had the advantages of a public school and university education, and whose parents have had to undertake the cost of such an education. These young men, by virtue of their liberal education, look at things from the broad and candid standpoint of a liberal and unconventional mental condition. They know how to reason philosophically; and whilst they are quite willing to admit that every profession is entitled to have its own code of obligation and honour, and that all who come into it ought to be prepared either to submit to the code or to show cause why they cannot do so, they are not willing to admit that a broad rule of freedom should be the privilege of seniors, and a narrow rule of injurious obligation should be forced upon juniors.

The question, then, comes to this in practice: Is it admissible or is it not admissible for medical writers to seek other than professional readers for their works? If it be admissible, then it must be admissible for all; if it be not admissible, then it must not be admissible for any. This position we can understand, and whichever way the whole profession decides to point in future, that way the whole of the honourable men among us will cheerfully walk. What the profession will not stand, and ought not to stand, is that an unwritten rule may be held up to terrorise the sensitive, and that the unscrupulous may set the rule at defiance, and that with the most absolute impunity.

